# A Deep Learning Method for Alerting Emergency Physicians about the Presence of Subphrenic Free Air on Chest Radiographs

**DOI:** 10.3390/jcm10020254

**Published:** 2021-01-12

**Authors:** Che-Yu Su, Tsung-Yu Tsai, Cheng-Yen Tseng, Keng-Hao Liu, Chi-Wei Lee

**Affiliations:** 1 Department of Emergency Medicine, Kaohsiung Medical University Hospital, Kaohsiung Medical University, Kaohsiung 80708, Taiwan; money0967@gmail.com; 2 Department of Medical Imaging, Kaohsiung Medical University Hospital, Kaohsiung Medical University, Kaohsiung 80708, Taiwan; saninbc@gmail.com; 3 Department of Mechanical and Electro-Mechanical Engineering, National Sun Yat-sen University, Kaohsiung 80424, Taiwan; he02162356@g-mail.nsysu.edu.tw; 4 Department of Physical Therapy, College of Health Science, Kaohsiung Medical University, Kaohsiung 80708, Taiwan; 5 Institute of Medical Science and Technology, National Sun Yat-sen University, Kaohsiung 80424, Taiwan

**Keywords:** hollow organ perforation, subphrenic free air, frontal chest X-ray images, emergency physicians, convolutional neural networks

## Abstract

Hollow organ perforation can precipitate a life-threatening emergency due to peritonitis followed by fulminant sepsis and fatal circulatory collapse. Pneumoperitoneum is typically detected as subphrenic free air on frontal chest X-ray images; however, treatment is reliant on accurate interpretation of radiographs in a timely manner. Unfortunately, it is not uncommon to have misdiagnoses made by emergency physicians who have insufficient experience or who are too busy and overloaded by multitasking. It is essential to develop an automated method for reviewing frontal chest X-ray images to alert emergency physicians in a timely manner about the life-threatening condition of hollow organ perforation that mandates an immediate second look. In this study, a deep learning-based approach making use of convolutional neural networks for the detection of subphrenic free air is proposed. A total of 667 chest X-ray images were collected at a local hospital, where 587 images (positive/negative: 267/400) were used for training and 80 images (40/40) for testing. This method achieved 0.875, 0.825, and 0.889 in sensitivity, specificity, and AUC score, respectively. It may provide a sensitive adjunctive screening tool to detect pneumoperitoneum on images read by emergency physicians who have insufficient clinical experience or who are too busy and overloaded by multitasking.

## 1. Introduction

Hollow organ perforation is one of the most common surgical emergencies, and its delayed diagnosis can be the cause of septic shock with multiple organ dysfunction syndrome. Delayed treatment of perforated peptic ulcer are major risk factors for complication and lethality [1,2,3]. A delay of more than 24 h increased mortality seven- to eight-fold and the complication rate three-fold [4]. Early diagnoses are crucial in the rescue of these patients, which heavily depends on the accurate interpretation of imaging studies [5].

The presence of free intraperitoneal air on chest or abdominal radiography most often provides the initial diagnostic impression of abdominal viscus perforation. Subphrenic free air results most commonly from perforated abdominal hollow organs, and a frontal chest radiograph is a standard method for its detection [6,7]. Pneumoperitoneum can be detected by radiography in 55 to 85% of patients with hollow organ perforation [8,9], though subphrenic free air is missed on frontal chest radiographs in 20 to 62% [10,11]. Although abdominal computed tomography (CT) is superior to frontal chest radiography in revealing subphrenic free air [11,12,13,14], it is not cost-effective for screening every single patient complaining of acute abdominal pain [10] and adds additional radiation exposure for the patient.

**Figure 1:**
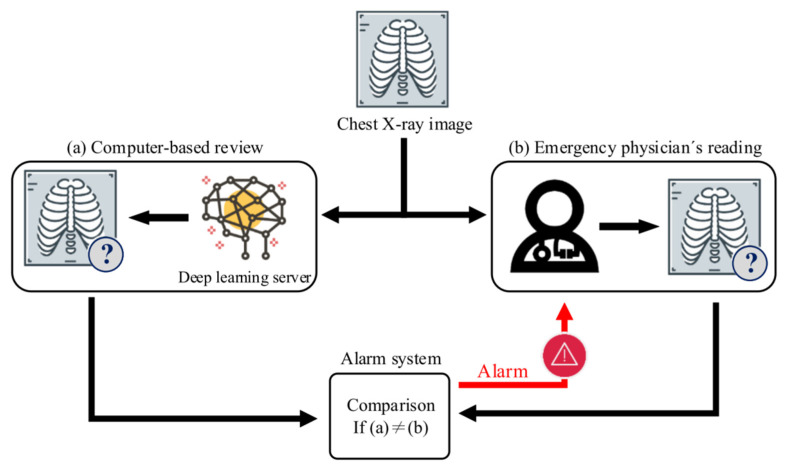
The concept of the automatic pneumoperitoneum alarm system. After a chest X-ray image of the patient is obtained, the image is sent to the deep learning server and the emergency physician for independent interpretation in parallel. If the computer-based review result is different from the emergency physician’s reading, the system will immediately send an alert message to the emergency physician.

Chest radiograph interpretation is a common practice of patient management in the emergency department (ED). Emergency physicians usually have to rely on themselves to make clinical decisions based on their own interpretation of chest radiographs because real-time overread by radiologists is not always available during office hours, let alone off hours. Unfortunately, misinterpretation is not uncommon when emergency physicians are overloaded by multitasking or when novice physicians lack sufficient experience. It is crucial to develop an automated method of reviewing frontal chest X-ray images to alert emergency physicians in a timely manner about the life-threatening condition of free hollow organ perforation that warrants, if not mandates, an immediate second look. The concept is shown in Figure 1.

Deep learning is an artificial intelligence method that can be used to recognize patterns that are useful in distinguishing images that reflect the presence or absence of a certain diseased lesion [15]. Recent advances in medical image analysis using such state-of-the-art technology have produced automated systems that can perform as well as, and even outperform, human experts in some medical tasks [16,17,18]. Sensitive and specific automation of subphrenic free air detection using X-ray studies would lead to earlier and more accurate diagnosis and hence, improve patient outcomes. Such automation would also reduce the need for expensive radiation CT studies, which could improve service efficiency and increase access to accurate detection of pneumoperitoneum in under-serviced regions. Automation could also improve reproducibility, given the reported variation in diagnostic certainty among human experts of different experience levels. The objective of this study is to create a large human-annotated dataset of frontal chest X-ray images containing subphrenic free air and to train deep convolutional neural networks (CNNs) through supervised learning to screen for subphrenic free air (dome sign) of pneumoperitoneum at the time of image acquisition in the emergency department.

## 2. Materials and Methods

This section first introduces the chest X-ray dataset for the study. The pre-processing for the dataset, the convolutional neural network (CNN) architecture, visualization process, and evaluation method are introduced later.

### 2.1. Due Diligence and Image Acquisition

Since ethical diligence remains our major concern, we endeavored to develop an artificial intelligence platform that could protect and prevent our patients from coming across misdiagnoses made by experienced but busy emergency physicians and inexperienced residents still under training.

**Figure 2:**
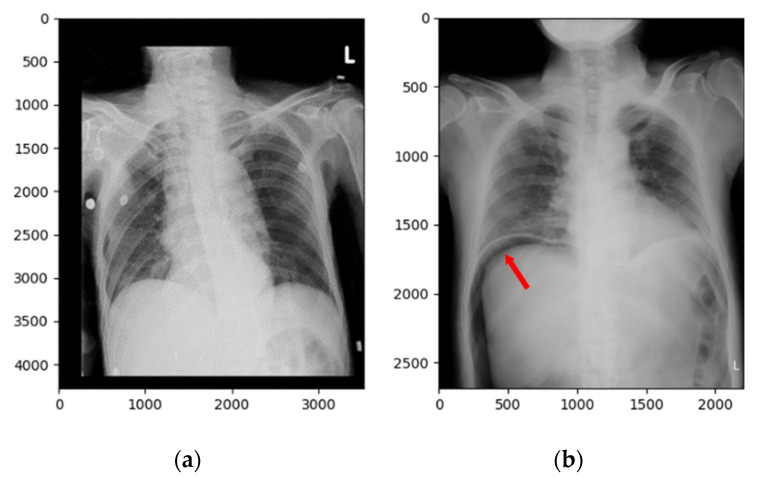
Frontal chest X-ray images of (**a**) a negative (normal) case and (**b**) a positive (pneumoperitoneum) case. The red arrow shows subphrenic free air, indicating pneumoperitoneum.

For technical diligence, evidence in the literature showed that the trained convolutional neural network fed with chest radiographs can actually meet the desired level of performance, and the amount of chest radiographs obtainable in the project made it feasible [18]. In addition, sufficient data for our study were obtained from the clinical radiology archive at Kaohsiung Medical University Hospital, a 1600-bed medical center in Taiwan, following approval granted by the Institutional Review Board (KMUHIRB-E-(II)-20190289). A total of 667 frontal chest X-ray images with and without subphrenic free air taken at the ED (annual census of around 96,000 patients per year) between 2007 and 2018 were included in the study. Initial pneumoperitoneum labels were obtained by combining the surgical operation records and findings from the radiology report archive. We collected one frontal chest X-ray image from each case with a total of 667 images. These were randomly divided into a training set (587 images) and a testing set (80 images) for model construction, with the ratio of positive and negative cases shown in Table 1. There was no overlapping of images between sets. The training set included only images referred from the ED, where lateral films and cross-sectional CT imaging are not routinely available in the first place and clinical decision-making is usually mandated prior to a formal radiology report. The prevalence of hollow organ perforation among the patients in the test set was 50%, whilst that among patients in the training set was lower (38.67%). Figure 2 shows two frontal chest X-ray image examples. One is from a normal image and the other is a pneumoperitoneum image.

Finally, regarding business diligence, the cost for obtaining one frontal chest X-ray image is only one-nineteenth that of a non-enhanced abdominal CT scan. Moreover, the whole body exposure dose of radiation in obtaining a frontal chest X-ray image is only one-thousandth that of an abdominal CT scan. Therefore, such a platform will drive value through lowering hospital costs by reducing the number of CT scans and compensation due to medicolegal penalties, and by squeezing more efficiency into the system in busy emergency rooms to alert physicians for a just-in-case second look. It turns out it is worthwhile developing such an automatic alarm system in terms of cost and safety.

### 2.2. Ground Truth Labeling for Hollow Organ Perforation

Hollow organ perforation is a promising target for deep learning approaches because of the availability of ground truth labels. Clinically, patients with hollow viscus perforation do not remain undetected. Because of the inevitable complication of peritonitis, initially “silent” perforation rapidly progresses to severe abdominal pain, rebound tenderness, abdominal muscle guarding, and hypotensive sequela.

In this study, all patients with hollow organ perforation that had imaging in the ED were identified in the radiology reports and the operative records. Our clinical experience suggests that the ground truth label accuracy is satisfactorily high.

### 2.3. Data Preparation

**Figure 3:**
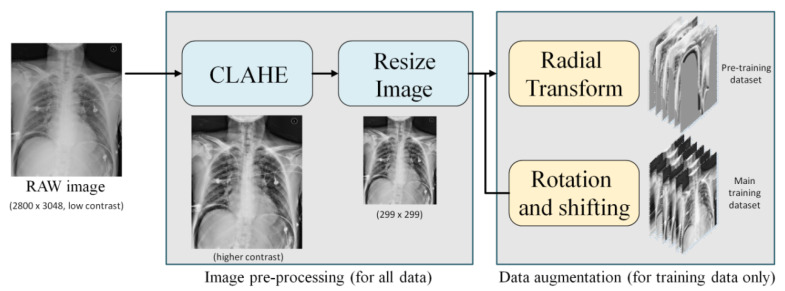
The flowchart of data preparation.

The data preparation included two steps: image preprocessing and data augmentation. Image preprocessing was applied to all X-ray images, while data augmentation was only applied to the training images. The procedure is shown in Figure 3.

In order to make it easier for the model to learn the features of the lesions and improve the test accuracy, we used contrast limited adaptive histogram equalization (CLAHE) [19] to enhance the contrast of the image. This can improve the over/underexposed regions within the image scene, and suppress the noise obtained in the image acquisition process. After applying CLAHE, each image was resized to 299 × 299.

In training a deep neural network, data augmentation is a routine step for better model robustness and less overfitting. In our study, two training datasets were organized: the pre-training dataset and the main training dataset.

- Pre-training dataset: To increase data diversity and enhance the relationship between adjacent tissues in the training process, for each raw image, we applied radial transform (RT) [20] to generate 6 augmented images to build a pre-training dataset. In total, this dataset includes 1362 positive samples and 2160 negative ones.
- Main training dataset: Each raw training image was augmented by using random horizontal shift ± 10%, vertical shift ± 10%, and rotation ± 10% to build the main training dataset. In total, it includes 1589 positive samples and 2520 negative ones.

### 2.4. Convolutional Neural Network (CNN)

Convolutional neural network (CNN) is a representative method in deep learning. With sufficient training material, CNN can learn the objective spatial features without any human-defined rules. CNNs have been widely used in image classification, object detection, and image segmentation. Recently, the applications of CNN performed on organ/lesion segmentation and image classification have caused great interest in the medical imaging community [21]. In this study, we selected three CNN models—Inception v4 [22], Inception-ResNet v2 [22], and DenseNet-121 [23]—to serve as the classifier of our method. They are briefly introduced as follows.

- Inception v4 is the improved version of Inception v3 [24]. It adds the Stem module to extract low-level features to improve the efficiency of using the Inception module [22]. It uses more Inception modules to strengthen the network’s ability to capture high-level features.
- Inception-ResNet v2 is composed of ResNet [25], the Inception network [26], and other units. ResNet makes the network converge faster and reduces the occurrence of gradient diffusion. The Inception module performs multiple convolution operations to obtain different levels of spatial information. All the outputs are concatenated to an output feature map with a large number of features. The other units are used for reducing the number of neurons or parameters.
- DenseNet-121 adopts Dense connections to improve the effect of feature reuse. In this architecture, each convolutional layer is connected with other convolutional layers through a feed-forward fashion. This design enables the model to reuse the features and reduce the issue of vanishing gradient.

### 2.5. CNN Training and Testing

**Figure 4:**
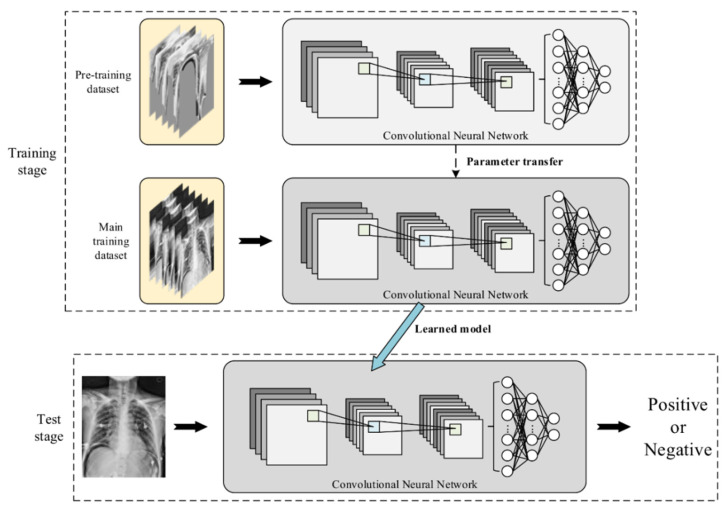
The training and test procedures of the proposed pneumoperitoneum classifier.

The overall model training and test procedure is shown Figure 4. The upper part indicates the training stage, while the lower part presents the test stage. In order to improve training speed and classification accuracy with insufficient training samples, we adopted a two-step training strategy, called transfer learning [27], to improve the discriminative ability and robustness of the model.

In the training stage, two identical CNN modules are used. The 1st training is performed with the Pre-training dataset on the upper module. The purpose is to make the model learn the relative relationship of the spatial characteristics between the lesion areas and normal ones. After the 1st training is completed, the learned parameters are transferred to the lower module as the initial condition of the 2nd training, which is carried out with the Main training dataset. Once the 2nd training is complete, the learned model will be used for test purpose.

In this experiment, we set the number of outputted classes to 2 (positive and negative). For training the hyperparameters, we set the batch number to 8. We chose cross entropy as the loss function for the softmax layer. The initial learning rate was set as $10^{-4}$ with the ADAM optimizer. For every two epochs, the learning rate will be reduced to 1/7. The training was stopped until 200 periods were finished. The whole process was implemented on Python with Tensorflow 1.13, under the hardware environment: Nvidia Geforce 1080 Ti, Intel i7-8700, and 32 GB DDR4 RAM.

Once the training stage was completed, the lower CNN module was then used as the pneumoperitoneum classifier for testing. Given a test image, the learned model outputs a continuous value between 0 and 1 that represents the probability that the image belongs to a positive sample. If it is greater than a predefined diagnostic cut-off threshold, the test image is classified as positive, otherwise negative. Based on this rule, all 80 test images were classified, the predicting results were collected, and a confusion matrix was established. In this paper, the diagnostic cut-off threshold is set to 0.5 as default.

### 2.6. Performance Metrics

To evaluate model effectiveness, five quantitative indices were used: Accuracy, Precision, Recall (Sensitivity), Specificity, and F1 Score. They can be obtained by using Equations (1)–(5), where TP, TN, FP, and FN denote the values of true positive, true negative, false positive, and false negative, respectively. The area under the ROC curve (AUC) is also used to illustrate the diagnostic ability of the proposed method.

(1) $$Accuracy\;=\;\frac{TP+TN}{TN+FP+FN+TP}$$

(2) $$Precision\;=\;\frac{TP}{FP+TP}$$

(3) $$Recall\;=\;Sensitivity\;=\;\frac{TP}{FN+TP}$$

(4) $$Specificity\;=\;\frac{TN}{TN+FP}$$

(5) $$F1\;Score\;=\;\frac{2}{\frac{1}{Precision}+\frac{1}{Recall}}$$

### 2.7. Visualization Method

**Figure 5:**
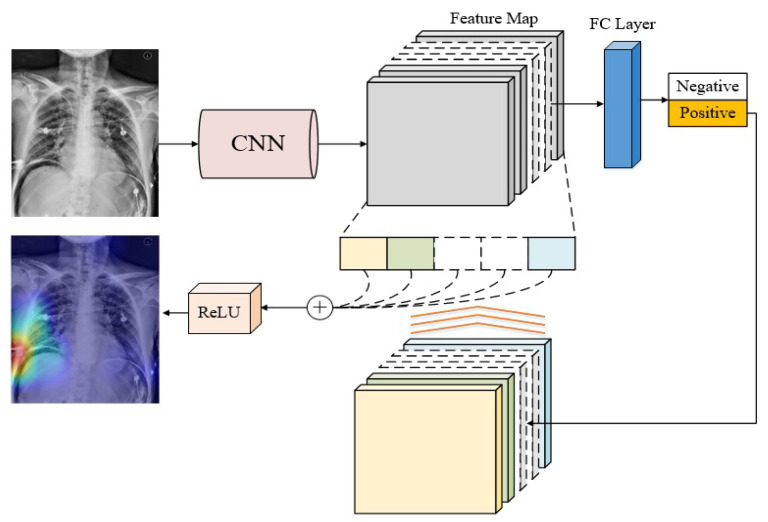
The diagram of Grad-CAM visualization method.

To validate whether the model has learned the lesion features correctly, Grad-CAM [28] is used to verify if the learned features from the positive samples are correlated with the lesion. Grad-CAM uses the weights of the feature map in the network to form a “heatmap” that highlights the areas where the classifier makes a positive decision. In other words, the heatmap emphasizes the lesion areas in each CT test image. Using Grad-CAM not only can help in hyperparameter tuning, but interpret the test results in a visual manner. The concept map of Grad-CAM is illustrated in Figure 5.

## 3. Results

### 3.1. Quantitative Performance

**Figure 6:**
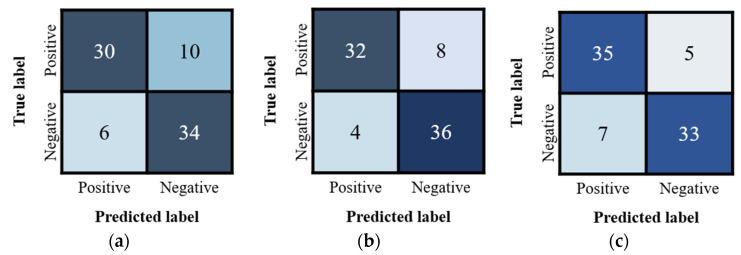
The confusion matrices of applying (**a**) Inception v4, (**b**) Inception-ResNet v2, and (**c**) DenseNet-121 on the 80 testing results.

For predicting the 80 test samples, the results of the confusion matrix are shown in Figure 6. It can be seen that using Inception-ResNet v2 and DenseNet-121 produced higher TP and TN as well as less FP and FN than using Inception v4.

The corresponding Accuracy, Precision, Recall, and F1 Score values are listed in Table 2. Similarly, it can be observed that in all aspects of quantitative metrics, using Inception-ResNet v2 and DenseNet-121 achieved significantly better performance than using Inception v4. Both Inception-ResNet v2 and DenseNet-121 achieved 0.85 in Accuracy, while Inception v4 barely achieved 0.8. The Inception-ResNet v2 performed better in Precision (0.889) and Specificity (0.9), while DenseNet-121 performed better in Sensitivity (0.875), F1 Score (0.853), and AUC (0.889). In conclusion, DenseNet-121 seemed to performance slightly better than Inception-ResNet v2.

**Figure 7:**
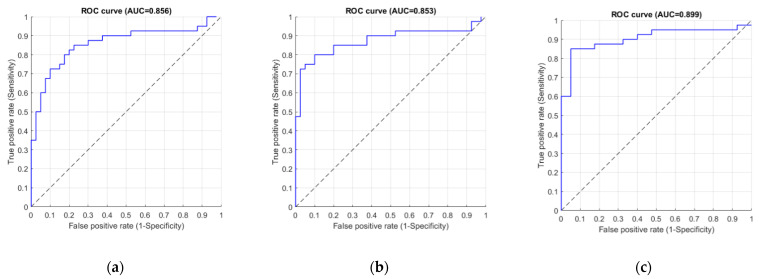
The ROC curves of different CNN models: (**a**) Inception v4 (**b**) Inception ResNet v2 (**c**) DenseNet-121. The *x*-axis false positive rate (or 1-specificity) and the *y*-axis denotestrue positive rate (or sensitivity). The dot line indicates the overall trend.

Moreover, the ROC curves of using three different CNNs are depicted in Figure 7. These ROC curves were drawn by varying the diagnostic cut-off threshold from 0 to 1, with an increment of 0.1. The corresponding AUC values are 0.856, 0.853, and 0.889, respectively. In this case, the maximum AUC was also achieved by using DenseNet-121.

### 3.2. Visualization

**Figure 8:**
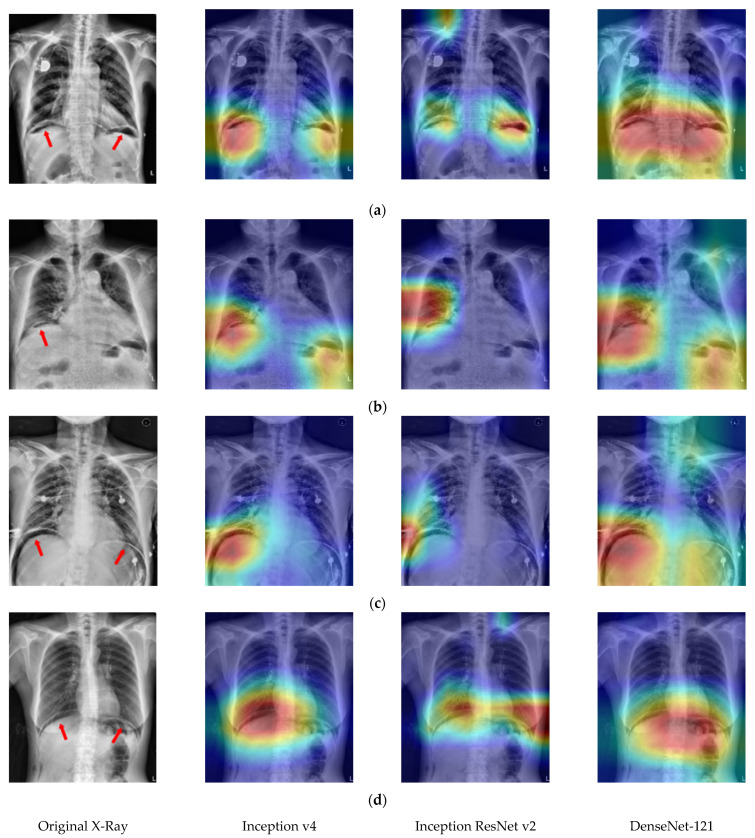
The heat map results of using three different CNNs on four selected TP cases (**a**–**d**). The red arrows in the first column indicate the lesion. The red areas in the 2nd to 4th columns are the regions where the CNN predicted them as positive cases.

In order to verify if the proposed CNN model uses the correct spatial features to make a decision, Figure 8 shows the Grad-CAM-produced heatmaps of four selected TP cases. In Figure 6a–c, the red regions (higher weights) almost appear on the lesions of PPU. This result implies that all three CNN models could learn the spatial relationship between lesions and the other normal tissues in the X-ray image.

## 4. Discussion

It is a common practice in most ED for attending emergency physicians to read the radiographs initially, followed by radiologists’ interpretations performed after the disposition of the patient [29,30,31,32]. Rapidly interpreting X-ray images to diagnose life-threatening conditions (such as hollow organ perforation) and then arrange appropriate surgical interventions immediately for patients is paramount to critical care, with direct impacts on the cost, efficiency, and quality of care of the ED. A competent emergency physician is expected to identify life-threatening lesions and take immediate actions on the first-hand findings of X-ray images prior to a radiologist’s review. Unfortunately, even experienced hands might make mistakes which end up with devastating consequences. As a matter of fact, we were inspired to run this study due to a recent experience of a medicolegal dispute due to a misdiagnosis event of a perforated peptic ulcer. An elderly male patient suffering from acute epigastralgia was admitted and sent to our ED. A frontal chest radiograph was taken and laboratory tests were performed for him. His abdominal pain was relieved after parenteral administration of analgesics. He was discharged from the ED soon after a review of the radiograph and laboratory test results. Subphrenic free air was identified in the chest radiograph by the radiologist during night shift duty. The emergency physician was notified about the abnormal finding immediately, and a phone call was made to a family member of the patient as soon as possible. Unfortunately, it was too late as the patient had already passed away at home.

Despite an overall high sensitivity and specificity of abdominal CT in the detection of pneumoperitoneum, it is expensive in terms of both radiative burden and cost. A non-enhanced abdominal CT scan is 19 times more costly than a frontal chest X-ray image in terms of monetary value. The effective radiation dose of a frontal chest X-ray image is about 0.01 mSv, whilst that of an abdominal CT scan is 10 mSv [33]. As such, abdominal CT is rarely used as an initial and routine diagnostic tool for abdominal pain. In this study, we endeavored to develop a deep learning algorithm that is capable of detecting pneumoperitoneum to fill the gap between chest X-ray images and abdominal CT. Our fully automated platform can take in any frontal chest X-ray and identify the presence of subphrenic free air automatically. As soon as the two-step training process based on a pre-training dataset and a main training dataset was completed, a learned model could then be achieved for further application in classifying a newly taken “pre-read” or “unknown” frontal chest X-ray image as either positive or negative. By means of the CLAHE algorithm, every single raw frontal chest X-ray image was enhanced and re-scaled to 299 × 299 to fit the input format of the neural network. Through data augmentation (transforming one image into six pre-training images by means of the technique of radial transform), a pre-training dataset of 3522 images in total was obtained. Furthermore, through data augmentation (by means of random horizontal shift ± 10%, vertical shift ± 10%, and rotation ± 10% of every single training image), a main training dataset of 4109 images in total was obtained. The area under our receiver operating characteristic curve (AUC) was found to be 0.889 in this study, which is considered to be satisfactorily high in terms of automated diagnosis. The outcome of this study demonstrates that despite the challenges specific to radiological image data, with the support of large and clean datasets, the construction of a highly automated deep learning alarm system for revealing hollow organ perforation on chest radiographs is achievable. Hopefully, the involvement of supervised machine learning technology could safeguard our patients from misdiagnosis of life-threatening problems due to erroneous decisions of novice emergency physicians or experienced but overloaded emergency consultants by alerting them for a second look of the radiographs that might warrant further investigations in a timely manner.

## 5. Limitations

There are some limitations in our study while using the proposed method. Firstly, the performance of a deep learning-based method relies on both data quantity and data quality. It is unlikely for the model to attain 100% accuracy. If the training materials are insufficient, the trained deep model is unable to make a decision accurately. Furthermore, using insufficient training data may lead to overfitting and reduce the generalization ability of the model. Compared with other deep learning applications related to medical imaging, the number of training images (i.e., total 667 images) we studied is relatively small. Therefore, the quantitative performance is limited.

**Figure 9:**
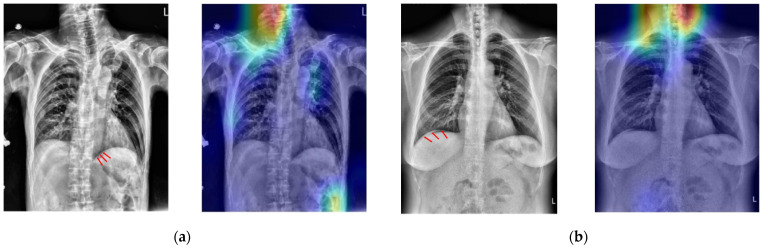
The raw X-ray images and the corresponding heatmaps of two FN cases (**a**,**b**). The deep model could not detect the small signs of pneumoperitoneum pointed out by the red arrows.

**Figure 10:**
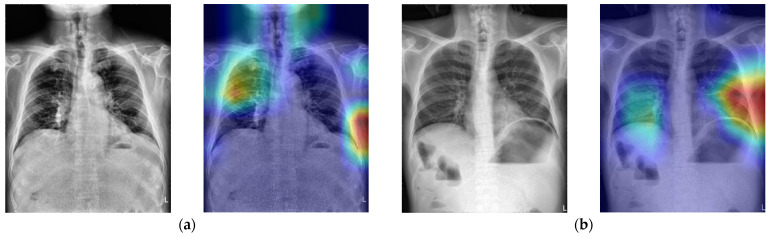
The raw X-ray images and the corresponding heatmaps of two FP cases (**a**,**b**). The deep model detected the “fake” dome signs that were caused by its contrast to intraluminal, rather than extraluminal, air of the gastrointestinal tract.

In addition to the quantity, the diversity of the data is another key. If the training dataset does not contain the rare cases of a particular symptom, the learned model will not recognize them. To explain this phenomenon, Figure 9 demonstrates the images of two examples of FN cases. Because most of the positive images in the training dataset have the free air under the diaphragm, which is considered as the representative feature of pneumoperitoneum, our trained model tended to use it to determine whether it has the symptom. Obviously, the raw CT images shown in Figure 9a,b do not contain subphrenic free air so that the trained CNN model could not identify them as positive. Similarly, Figure 10 demonstrates the images of the two FN cases. These images have characteristics similar to subphrenic free air under the right diaphragm, which makes the classifier misjudged them as positive. Therefore, in order to build a reliable pneumoperitoneum alarm system in the future, it is necessary to collect more training materials that contain images of diverse symptoms for model training.

Furthermore, at this stage, the sensitivity of this approach is barely 87.5% (DenseNet-121); improvement is mandatory to make it a trustworthy tool for alerting emergency physicians to have a second look at the chest X-ray film. Otherwise, multiple experiences of false negative readings produced by the system might give an impression of “unreliability” to the busy physicians and end up in abandoning of further use. Moreover, the specificity of this approach is barely 82.5% (DenseNet-121); refinement is imperative to make it a useful tool for alerting emergency physicians to have a second look at the chest X-ray film. Otherwise, multiple experiences of false positive reading produced by the system might give an impression of “redundancy” and “a waste of time” to the busy physicians and cause them to give up further application of the system. These limitations should be improved in the future in order to achieve higher sensitivity and specificity of the proposed alarm method.

## 6. Conclusions

Hollow organ perforation is one of the most common surgical emergencies, and its delayed diagnosis can be the cause of septic shock with multiple organ dysfunction syndrome. In this study, a deep learning method is proposed to detect subphrenic free air in frontal chest X-ray films at high levels of performance on held-out test data. This method is composed of a preprocessing procedure, and a CNN-based classification network trained under a two-step strategy. The experiments conducted on 667 frontal chest X-ray images demonstrate that the method achieved satisfactory results in sensitivity, specificity, and AUC values. It may provide a sensitivity adjunctive screening tool to detect pneumoperitoneum on frontal chest X-ray images read by emergency physicians who have insufficient clinical experience or by emergency physicians who have sufficient experience but who are too busy and overloaded by multitasking. They are not intended to replace human expert review of frontal chest X-ray images for pneumoperitoneum. Development of a practical deep learning algorithm is worthwhile to develop appropriate, effective clinician alerts for the potentially critical finding of free hollow organ perforation, and to reducing the risk of misdiagnosis.

## Figures and Tables

**Table 1:** Train–test split for the chest X-ray dataset.

	Training Dataset	Test Dataset
Positive (Pneumoperitoneum)	227	40
Negative (Normal)	360	40
Total	587	80

**Table 2:** The quantitative results of using different convolutional neural networks (CNNs).

	Inception v4	Inception Resnet v2	DenseNet-121
Accuracy	0.800	**0.850**	**0.850**
Precision	0.833	**0.889**	0.833
Sensitivity	0.750	0.800	**0.875**
Specificity	0.850	**0.900**	0.825
F1-Score	0.789	0.842	**0.853**
AUC	0.856	0.853	**0.899**

The bold values indicates the best performance in each metric.

## Data Availability

The data presented in this study are available in this published article titled “A deep learning method for alerting emergency physicians about the presence of subphrenic free air on chest radiographs.”
